# Synthesis and Biological Activity of Antimicrobial Agents

**DOI:** 10.3390/antibiotics11030337

**Published:** 2022-03-04

**Authors:** M. Fernanda N. N. Carvalho

**Affiliations:** Centro de Química Estrutural, Institute of Molecular Sciences Instituto Superior Técnico, Universidade de Lisboa, Av. Rovisco Pais, 1049-001 Lisboa, Portugal; fcarvalho@tecnico.ulisboa.pt

New antimicrobial agents are urgent and necessary to overcome the acquired resistance of microorganisms to existing antibiotics and antifungals. In the end of 2021, the World Health Organization (WHO) declared antibiotics microbial resistance (AMR) as one of the ten global human health threats [1]. The European Commission estimates that in the European Union, there are 33,000 deaths per year due to AMR and the costs due to healthcare and productivity losses achieve EUR 1.5 billion [2]. According to predictions, by 2050, if no action is taken, the worldwide mortality due to AMR will reach 10 million per year and the related costs ca. USD 1 trillion [3]. Such figures clearly show the need for new efficient antimicrobial agents able to treat infections and overcome microorganisms (bacteria or fungi) resistance. Aware of this, the scientific community engaged in the study of strategies to discover efficient antimicrobials that act through mechanisms different from those in use to fight infections and overcome the acquired microbial resistance. One of the strategies is focused on the synthesis of new compounds, either coordination or organic, and another on the preparation of nanoparticles and assessment of their corresponding antimicrobial properties. Examples of each one of such approaches exist in the Special Issue *Synthesis and Biological Activity of Antimicrobial Agents* in the MDPI *Antibiotics* that comprises two reviews and nine research papers on the synthesis of organic or coordination compounds and nanoparticles, either bare or coated. 

M. Marinescu [4] reviews the methodologies to synthesize benzimidazole–pyrazole compounds and organizes their reported antibacterial and antifungal properties, also mentioning the analgesic and anticancer properties of some of them. In conclusion, M. Marinescu highlights the relevance for the biological activity of the position of the pyrazole substituent at the benzimidazole skeleton, the characteristics of the linker between the two units and the beneficial effect of selected substituents at the pyrazole or the benzimidazole moieties. In another report based on new organic compounds, R. Schobert et al. [5] describe a synthetic route to obtain versatile precursors for polycyclic tetramic acid macrolactams with structural analogies to macrocidins A and Z. Eight new organic compounds were reported with herbicidal, antibiotic and/or antibiofilm activities in some cases pointing to feasible low induction of bacterial resistance. The synthesis of fifteen acetylene substituted dihydropyrrolones (DHPs) is reported by N. Kumar et al. [6] based on the lactamisation and Sonogashira coupling reactions. The compounds were tested for a quorum-sensing inhibitory (QSI) effect against *P. aeruginosa*, showing moderate activity towards the inhibition of signalizing molecules produced by bacteria. 

In what concerns new antimicrobial agents based on coordination compounds, J. Leitão et al. and M. Alves et al. report studies involving silver and gold camphorimine complexes. J. Leitão et al. [7] describe the synthesis, structural characterization, and antibacterial properties of Ag(I) and Au(I)) camphorimine complexes. The moderate-to-high activity of the complexes varies with the substituents on camphorimine ligands. In the case of Au(I) complexes, a correlation was found between the MIC values and the gold content concerning the antibacterial activity towards *B. contaminans* and *P. aeruginosa* strains. M. Alves et al. [8] report the application of selected Ag(I) camphorimine complexes as antifungal on the functionalization of PCL for stainless steel coating, showing that the complexes do not lose activity and effectively inhibit biofilm formation by *C. albicans*. 

The synthesis and characterization of nanoparticles is a developing research domain. In this Special Issue, six out of the eleven contributions deal with the synthesis of nanoparticles designed towards biological applications. Three of them, focused on cost-effective, green, and eco-friendly methods, describe the biosynthesis of nanoparticles using leaf extracts or bacillus. The biosynthesis of platinum [9] and gold [10] nanoparticles using the leaf extract of *Combretum erythrophyllum* (CE) is reported by O.S. Oluwafemi et al. The water-soluble, spheric, very small (average diameter 1.04 ± 0.26 nm) platinum nanoparticles (PtNPs) were characterized by different techniques. PtNPs are biologically active against Gram-positive *Staphylococcus epidermidis* (ATCC 14990) and Gram-negative *Klebsiella* strains. The CE-stabilized PtNPs show enhanced selectivity towards Klebsiella strains that cause nosocomial infections. Platinum and gold nanoparticles (AuNPs), for example, were morphologically characterized. They are spherical, well dispersed, and slightly bigger than PtNPs. When probed for biological activity, AuNPs showed moderate activity towards selected Gram-positive and Gram-negative bacteria and high biocompatibility with cancer (cervical Hela and lung A549) and normal (BHK-21) cell lines. A. Khan and M. Mathanmohu [11] reported the synthesis of silver nanoparticles (AgNPs) using *Bacillus* mn14. The AgNPs were fully characterized (UV/VIS, XRD, SEM, AFM), and their antifungal activity towards selected fungi spp evaluated. AgNPs revealed excellent antagonistic activity towards the human pathogen *S. viridans* and beneficial effects in the repair of sick leaves and growth of shoots and roots of a rice plant. 

In addition to potential direct biological activity, applications of nanoparticles include their use as drug deliverers by coating with antimicrobials. Such an ability is explored by D. R. Monteiro et al. [12] to deliver drugs in use (miconazole or fluconazole) to biofilms formed by *C. albicans* and *C. glabrata*. The iron oxide nanoparticles (IONPs-CS) used as drug carriers were obtained through chitosan coating of iron oxide nanoparticles. The *Candida* biofilms were characterized and their inhibition by IONPs-CS evaluated, showing that the process effectively works and that the activity of the coated IONPs-CS in some cases surpass that of the free antifungals. Bioactive nanoparticles with magnetic properties enable applications such as imaging or theranostics [13] not viable for other types of bioactive nanoparticles. Magnetic small-sized (average diameter 16 nm) cationic nanoparticles were synthesized by D. Horák et al. [14] via thermal decomposition of Fe (III) oleate and coating with Sipomer PAM-200. Their characteristics were studied by size-exclusion chromatography (SEC), transmission electron microscopy (TEM), and dynamic light scattering (DLS), among other techniques. The evaluation of the antibacterial properties of these cationic polymer-coated magnetite nanoparticles against *E. coli* and *S. aureus* showed that they have higher activity than the related non-magnetic iron oxide nanoparticles. Finally, in the review “Bacterial and virucidal activities of biogenic metal-based nanoparticles”, G. Tortella et al. [15] address the recent advances and perspectives of biogenically synthesized nanoparticles, highlighting the variety of species that can be used as reducing agents. These reducing agents affect the morphology and aggregation of the nanoparticles and consequently their biological activity. Among the addressed types of biogenically synthesized nanoparticles, details are given concerning the antimicrobial activity of AgNPs, CuNPs, ZnO-NPs, TiO_2_-NPs, FeO-NPs, NiO-NPs, Pd-NPs, and SnO_2_-NPs species. G. Tortella et al. advertise that care is necessary in selection of the reducing agents due to the toxicity of some microorganisms, algae, and plants. They also point out the need for better strategies to control the size distribution, morphology, and chemical surface of nanoparticles. 

In this Special Issue of the MDPI journal *Antibiotics*, several different approaches exist on the way to new efficient antimicrobials. Considering the contributions to *Synthesis and Biological Activity of Antimicrobial Agents*, a step forward would be the combination of new drug-delivery nanocarriers with new active compounds (either or organic or coordination) acting through mechanisms different from the drugs in use.

## References

[1] WHO (2021). Antimicrobial Resistance. https://www.who.int/news-ro]om/factsheets/detail/antimicrobial-resistance.

[2] EU Action on Antimicrobial Resistance. https://ec.europa.eu/health/antimicrobial-resistance/eu-actionantimicrobial-resistance_en.

[3] Dadgostar P. (2019). Antimicrobial Resistance: Implications and Costs. Infect. Drug Resist..

[4] Marinescu M. (2021). Synthesis of Antimicrobial Benzimidazole–Pyrazole Compounds and Their Biological Activities. Antibiotics.

[5] Treiber L., Pezolt C., Zeng H., Schrey H., Jungwirth S., Shekhar A., Stadler M., Bilitewski U., Erb-Brinkmann M., Schobert R. (2021). Dual Agents: Fungal Macrocidins and Synthetic Analogues with Herbicidal and Antibiofilm Activities. Antibiotics.

[6] Almohaywi B., Yu T.T., Iskander G., Sabir S., Bhadbhade M., Black D.S., Kumar N. (2022). Synthesis of Alkyne-Substituted Dihydropyrrolones as Bacterial Quorum-Sensing Inhibitors of *Pseudomonas aeruginosa*. Antibiotics.

[7] Costa J.P., Sousa S.A., Soeiro C., Leitão J.H., Galvão A.M., Marques F., Carvalho M.F.N.N. (2021). Synthesis and Characterization of Camphorimine Au(I) Complexes with a Remarkably High Antibacterial Activity towards *B. contaminans* and *P. aeruginosa*. Antibiotics.

[8] Pinheiro M., Costa J., Marques F., Mira N., Carvalho M., Alves M. (2021). Bioactive Coatings with Ag-Camphorimine Complexes to Prevent Surface Colonization by the Pathogenic Yeast *Candida albicans*. Antibiotics.

[9] Fanoro O.T., Parani S., Maluleke R., Lebepe T.C., Varghese R.J., Mgedle N., Mavumengwana V., Oluwafemi O.S. (2021). Biosynthesis of Smaller-Sized Platinum Nanoparticles Using the Leaf Extract of Combretum erythrophyllumand Its Antibacterial Activities. Antibiotics.

[10] Fanoro O., Parani S., Maluleke R., Lebepe T., Varghese J., Mavumengwana V., Oluwafemi O. (2021). Facile Green, Room-Temperature Synthesis of Gold Nanoparticles Using *Combretum erythrophyllum* Leaf Extract: Antibacterial and Cell Viability Studies against Normal and Cancerous Cells. Antibiotics.

[11] Kabeerdass N., Al Otaibi A., Rajendran M., Manikandan A., Kashmery H.A., Rahman M.M., Madhu P., Khan A., Asiri A.M., Mathanmohun M. (2021). *Bacillus*-Mediated Silver Nanoparticle Synthesis and Its Antagonistic Activity against Bacterial and Fungal Pathogens. Antibiotics.

[12] Caldeirão A.C.M., Araujo H.C., Tomasella C.M., Sampaio C., dos Santos Oliveira M.J., Ramage G., Pessan J.P., Monteiro D.R. (2021). Effects of Antifungal Carriers Based on Chitosan-Coated Iron Oxide Nanoparticles on Microcosm Biofilms. Antibiotics.

[13] Gul S., Khan S.B., Rehman I.U., Khan M.A., Khan M.I. (2019). A Comprehensive Review of Magnetic Nanomaterials Modern Day Theranostics. Front. Mater..

[14] Shatan A.B., Patsula V., Dydowiczová A., Gunár K., Velychkivska N., Hromádková J., Petrovský E., Horák D. (2021). Cationic Polymer-Coated Magnetic Nanoparticles with Antibacterial Properties: Synthesis and In Vitro Characterization. Antibiotics.

[15] Tortella G., Rubilar O., Fincheira P., Pieretti J., Duran P., Lourenço I., Seabra A. (2021). Bactericidal and Virucidal Activities of Biogenic Metal-Based Nanoparticles: Advances and Perspectives. Antibiotics.

